# Supplementary material for: Identification of photoperiod-induced specific miRNAs in the adrenal glands of Sunite sheep (Ovis aries)
Source: Front Vet Sci. 2022 Jul 22;9:888207. doi: 10.3389/fvets.2022.888207 (PMC9354845; doi:10.3389/fvets.2022.888207)
Supplement: Supplementary file 1 [file Data_Sheet_1.ZIP › Supplementary file/miRDeep2 file.zip!/miRDeep2 file/Know_Structure/SP42A1/oar-mir-539.pdf]

miRBase precursor : oar-mir-539
Total read count : 894
oar-miR-539-5p read count : 4
oar-miR-539-3p read count : 890
remaining reads : 0

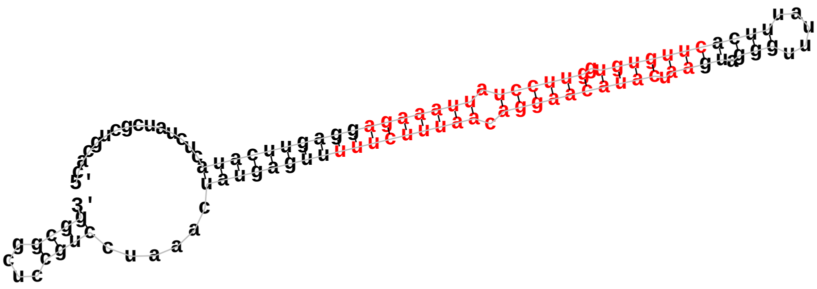

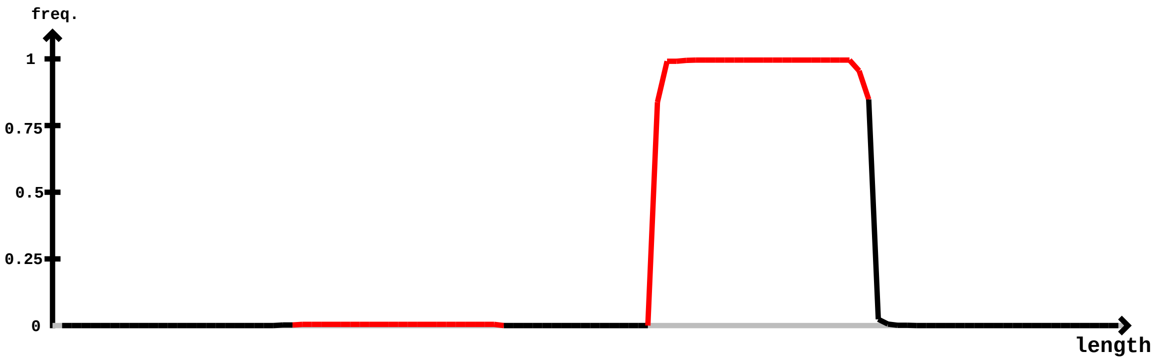


```
                                                                        oar-miR-539-3p
                                    oar-miR-539-5p
5'- cacgucgcuaucucauacuugaggagaaauuauccuugguguguucacuuuauuugggaugaaucauacaaggacaauuucuuuuugaguaucaaauccugccucggcgg   -3'   exp
    ..............(((((((((((((((((.((((((.(((((((((((.....))).)))).)))))))))).)))))))))))))))))......((((....))))   reads   mm   sample
    ......................ggagaaauuauccuugguguguu.................................................................   2       0    seq
    ........................agaaauuauccuugguguguu.................................................................   2       0    seq
    .............................................................aaucauacaaggacaauuucu............................   36      0    seq
    .............................................................aaucauacaaggacaauuucuu...........................   89      0    seq
    .............................................................aaucauacaaggacaauuucuuu..........................   616     0    seq
    .............................................................aaucauacaaggacaauuucuuuu.........................   8       0    seq
    ..............................................................aucauacaaggacaauuucu............................   7       0    seq
    ..............................................................aucauacaaggacaauuucuuu..........................   121     0    seq
    ..............................................................aucauacaaggacaauuucuuuu.........................   8       0    seq
    ..............................................................aucauacaaggacaauuucuuuuu........................   1       0    seq
    ................................................................cauacaaggacaauuucuuuuu........................   3       0    seq
    .................................................................auacaaggacaauuucuuuuuga......................   1       0    seq
```
